# The Brave study: promoting active breaks in secondary school from students’ point of view: Matteo Ricci

**DOI:** 10.1093/eurpub/ckac131.454

**Published:** 2022-10-25

**Authors:** M Ricci, A Masini, G Longo, A Sansavini, LM Scheier, S Marini, A Ceciliani, L Dallolio

**Affiliations:** 1 Department of Biomedical and Neuromotor Sciences, Alma Mater Studiorum - University of Bologna, Bologna, Italy; 2 Department of Psychology “Renzo Canestrari”, Alma Mater Studiorum - University of Bologna, Bologna, Italy; 3 LARS Research Institute, Inc., Scottsdale, USA; 4 Department for Life Quality Studies, Alma Mater Studiorum - University of Bologna, Rimini, Italy

## Abstract

**Background:**

According to the WHO recommendations, children and adolescents should perform at least 60 minutes per day of moderate to vigorous physical activity (PA). Active Breaks (ABs) interventions, short physical activity breaks of 5-15 minutes during school hours, have been examined in primary school children as a potential strategy to counteract a sedentary lifestyle, with minimal disruption to school learning activities. The aim of the BRAVE STUDY is to investigate the feasibility of ABs in a secondary school setting from the students’ point of view.

**Methods:**

In December 2020, 10 students (age 12-13, 6 females and 4 males) attending the second and third grade of secondary schools located in Bologna province (Italy) were involved in a focus group (FG). The FG was conducted online because of COVID-19 and the answers transcribed for a later analysis. Students’ opinions were probed on the role of PA in school and possible facilitators/barriers to implementation of ABs in the school.

**Results:**

Students reported they wanted to be more active as a consequence of time spent in class sitting at a desk. Students also reported that an organized activity like ABs conducted in the classroom setting provides an excellent opportunity to improve social relations with classmates. Students also highlighted the possible psycho-physical well-being benefits arising from PA. Among the potentially negative aspects reported, students underlined the possible confusion that would be created in classroom and the time subtracted from academic learning activities.

**Conclusions:**

The FG represents an ideal mean to obtain in-depth information on how people feel about a pending program or a change in their routine. The current FG reinforces positive outcomes from exposure to a PA program that can be intertwined with their daily classroom activities. ABs programs can help to reconcile the needs of students that arise during the day with the PA objectives recommended by the WHO.

**Key messages:**

• ABs can be a zero-cost intervention strategy to achieve WHO recommendations and would create conditions for a greater psycho-physical benefits in classrooms.

• A qualitative approach, such as FGs, provides a mean to collect information not obtainable with quantitative methods, that could be useful to co-design interventions for children and adolescents.

